# Improved radiotracing of oxytocin receptor-expressing tumours using the new [^111^In]-DOTA-Lys^8^-deamino-vasotocin analogue

**DOI:** 10.1038/sj.bjc.6601189

**Published:** 2003-08-26

**Authors:** B Chini, M Chinol, P Cassoni, S Papi, A Reversi, L Areces, T Marrocco, G Paganelli, M Manning, G Bussolati

**Affiliations:** 1 CNR Institute of Neuroscience, Cellular and Molecular Pharmacology Section, Via Vanvitelli 32, 20129 Milano, Italy; 2 European Institute of Oncology, Division of Nuclear Medicine, Via Ripamonti 435, 20141 Milano, Italy; 3 Department of Biomedical Sciences and Human Oncology, University of Turin, Via Santena 7, 10126 Torino, Italy; 4 Department of Biochemistry and Molecular Biology, Medical College of Ohio, Toledo, PO Box 10008, OH, USA

**Keywords:** oxytocin receptor, tumours, radioligand, OT, LVT

## Abstract

Oxytocin receptors (OTR) have been described in a number of tumours of different origin, and represent a new target for specific radiolabelled oxytocin (OT) analogues in cancer diagnosis and therapy. By linking the DOTA chelating agent to position 8 of the deamino derivative of Lys^8^-vasotocin (dLVT), we obtained a new compound (DOTA-dLVT) with the following characteristics: (1) it forms a monomeric and stable compound that binds to OTR with an affinity comparable to that of the endogenous OT ligand; (2) it is characterised by a very good selectivity profile for the human OTR, with a low affinity binding to the closely related V1a, V1b and V2 vasopressin receptor subtypes; (3) it induces rapid and persistent receptor internalisation and (4) when radiolabelled, [^111^In]-DOTA-dLVT is efficiently and selectively taken up by OTR-positive tumours grown in mice. These features makes radiolabelled DOTA-dLVT a very good candidate for the radiotargeting of OTR-expressing tumours.

The observation that a variety of tumours overexpress natural peptide receptors has served as the molecular basis for the clinical use of radiolabelled peptides in peptide receptor radionuclide therapy (PRRT) (de Jong and Krenning, 2002), a strategy that has already proved to be successful in detecting and treating somatostatin receptor-positive tumours using radioactive somatostatin or its analogues (Froidevaux and Eberle, 2002). In these tumours, the heavy deposition of radioactive metals such as [^111^In] and [^90^Y], which leads to valuable radiodetection and radiotherapeutic effects, was achieved using the 1,4,7,10-tetraazacyclododecane-*N*,*N*′,*N*″,*N*′″-tetraacetic acid (DOTA) chelating agent irreversibly bound to a free amino group of somatostatin or its analogues (Merlo *et al*, 1999).

In order to apply PRRT to tumours lacking somatostatin receptors, other natural peptides need to be investigated. The oxytocin (OT) hypothalamic nonapeptide binds to various tumour types via the oxytocin receptor (OTR), a member of the seven transmembrane spanning family of G-protein-coupled receptors (Kimura *et al*, 1992; Gimpl and Fahrenholz, 2001). The tumoral expression of OTRs was first described in human breast cancer cell lines (Cassoni *et al*, 1994) and a large percentage of primary breast cancers (Bussolati *et al*, 1996). OTRs have recently also been detected in tumours of the nervous system such as neuroblastomas and glioblastomas (Cassoni *et al*, 1998), osteosarcomas (Novak *et al*, 2000), chorioncarcinomas (Cassoni *et al*, 2001), small cell lung carcinomas (Pequeux *et al*, 2002) and Kaposi sarcomas (Cassoni *et al*, 2002). It can therefore be hypothesised that suitable OT analogues appropriately labelled with a radionuclide may be useful in the diagnosis and treatment of a number of tumours arising from different tissues. Furthermore, it has also recently been shown that the human OTR in breast cancers is present in a cluster of overexpressed genes related to intrinsic and acquired doxorubicin resistance, and therefore represents a potential therapeutic target for the management of chemoresistant breast tumours (Turton *et al*, 2001).

Interest in OTR expression in such a large range of tumour types has increased since the recent development of a novel compound potentially suitable for radioreceptor-mediated diagnosis and therapy (Bussolati *et al*, 2001). This compound was obtained by linking an OT analogue ([Lys8]-vasotocin; LVT) to the DOTA chelating agent, which is capable of efficaciously binding ^111^In and ^90^Y. In LVT, the leucine in position 8 of OT is replaced by a lysine, thus providing a suitable binding site for the DOTA molecule. DOTA-LVT has proved to be highly efficient in linking ^111^In and transferring the radioactive metal to OTR-positive breast cancer cells *in vitro* and *in vivo* in tumour-bearing mice. However, because of the reactivity of the free amino group of cysteine in position 1 of LVT, polymeric complexes were formed during the process of DOTA conjugation, and the final peptide mixture had low binding affinity for the human OTR (from 10^−9^ to 10^−7^ M).

The aim of the present study was to develop an improved radiotracer characterised by high affinity and specificity for OTR-expressing tumours. To this end, we used a deaminated LVT at position 1 (dLVT) (Rimpler, 1971) for the synthesis of a monomeric radiotracer ([^111^In]-DOTA-dLVT). This new radiotracer not only retained high and specific affinity for OTR and OTR-positive cells *in vitro* and *in vivo*, but was also efficiently taken up by OTR-positive tumours *in vivo*. [^111^In]-DOTA-dLVT thus represents a potentially powerful new reagent for the imaging and treatment of OTR-positive tumours.

## MATERIALS AND METHODS

### Reagents

DOTA,4H_2_O (MW of the dehydrated form: 404.4) × 4H_2_O was purchased from Macrocyclics (Richardson, TX, USA), OT, LVT (MW 1022.2) (Boissonnas and Huguenin, 1960) and dLVT (MW 1007.2) (Rimpler, 1971) were resynthesised as previously described (Terrillon *et al*, 2002). ^111^In Cl_3_ was obtained from Mallinckrodt Medical (Petten, The Netherlands); [^3^H]OT (35–45 Ci mmol^−1^) and [^3^H]AVP (35–45 Ci mmol^−1^) were from NEN Life Science Products (Boston, USA); the other chemicals were purchased from Sigma (St Louis, MO, USA).

### Plan of the experiments

To prove the efficient binding of the radioactive OT analogue to OTR-positive tumour cells, we first had to link the DOTA chelating agent to dLVT and show that the resulting compound (DOTA-dLVT) was monomeric and still retained affinity for OTR. We then radiolabelled DOTA-dLVT with ^111^In and tested the binding of [^111^In]-DOTA-dLVT to OTR-positive cells and tumours *in vitro* and *in vivo*.

### Conjugation of DOTA to dLVT

**Figure 1:**
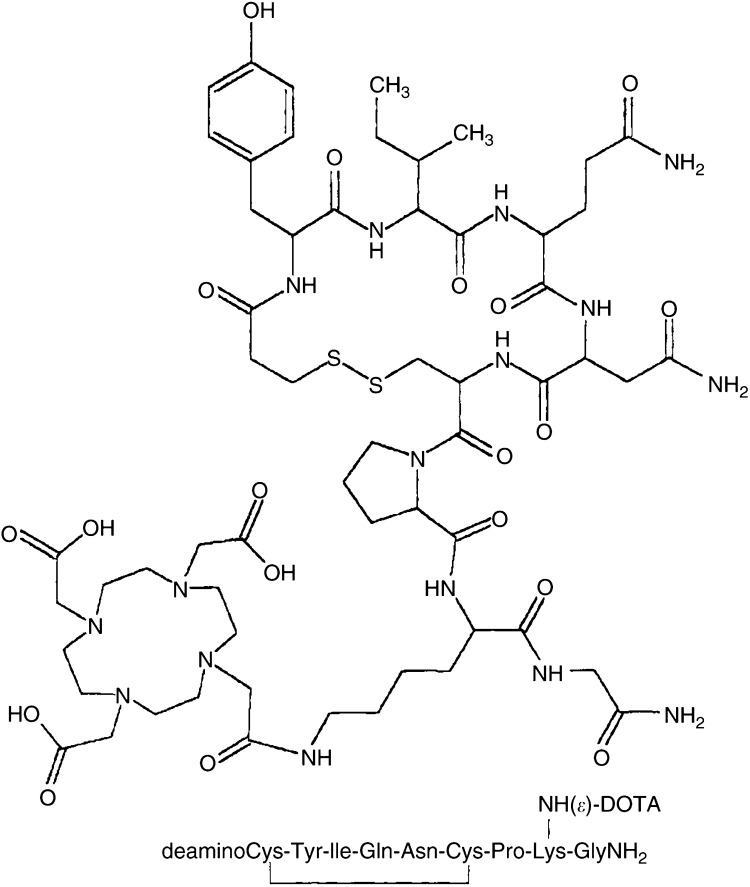
Structure of conjugate DOTA-dLVT. Owing to the lack of the Cys^1^ amino group, the DOTA moiety is selectively linked to the only available *ɛ*-NH_2_ of Lys^8^.

**Figure 2:**
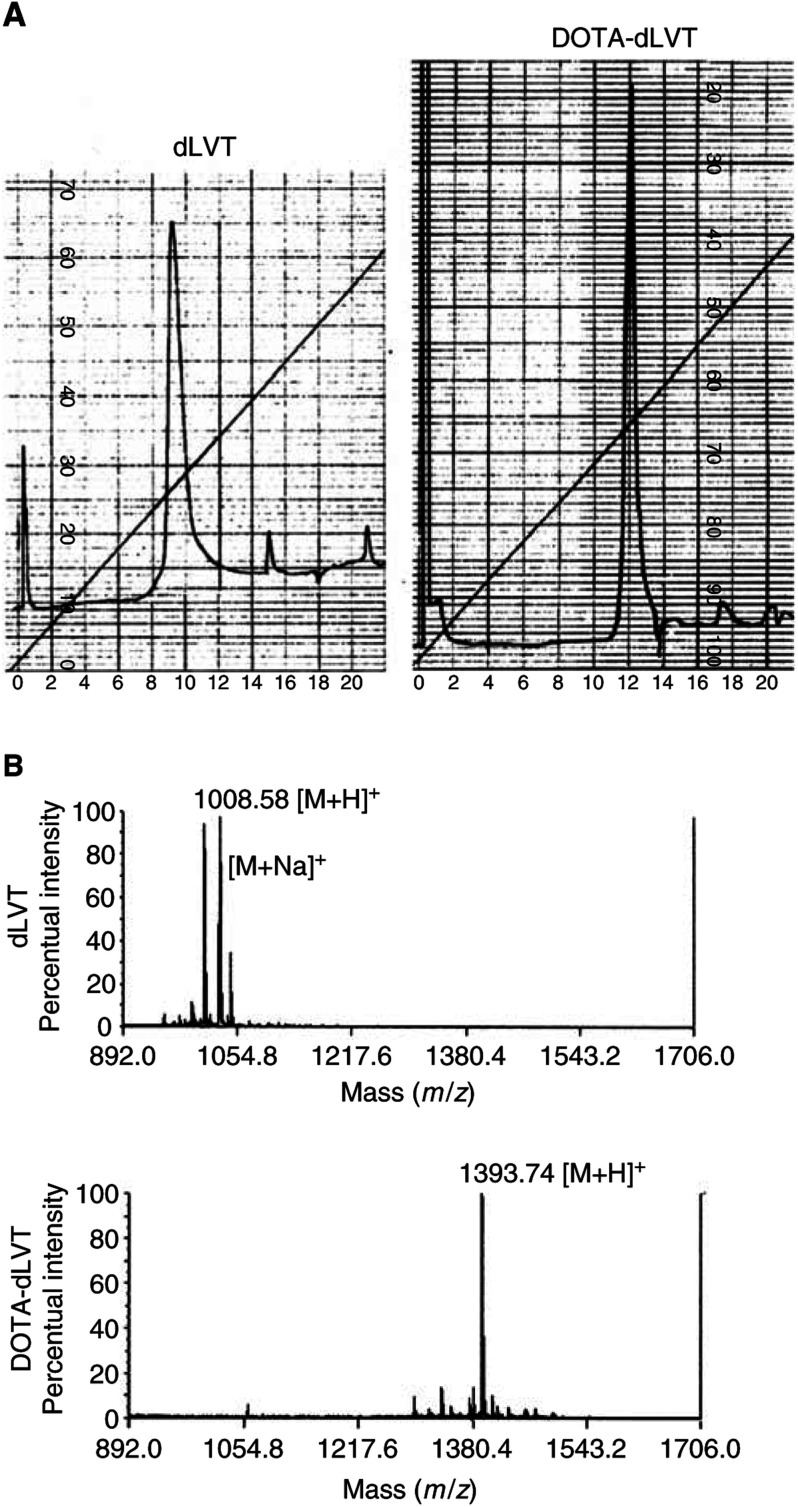
Panel (**A**): FPLC profile of dLVT and DOTA-dLVT (*λ*=280 nm, AUFS=0.1 for both chromatographic runs). The starting peptide dLVT had a retention time of 9 min (minor peaks are due to pressure fluctuations). After DOTA conjugation, the DOTA-dLVT peak was detected after 12 min. Panel (**B**): MALDI-TOF analysis of dLVT and DOTA-dLVT after purification. Mass spectra of the two compounds show that conjugation has successfully taken place, giving rise to the desired product DOTA-dLVT. The second peak in the dLVT spectra is due to the presence of some residual Na^+^ ions after sample desalting ([M+Na]^+^).

The COOH group of DOTA was activated by means of a carbodiimide reagent (Buckley and Searle, 1984). Briefly, DOTA was dissolved in anhydrous dimethyl sulphoxide (DMSO) at 80°C and the solution was allowed to cool to room temperature under an argon atmosphere. A solution of *N*-hydroxy-2,5-pyrrolidinedione (*N*-hydroxysuccinimide; NHS) in DMSO was added dropwise to the stirring solution of DOTA, followed by the dropwise addition of *N*,*N*′-dicyclohexylcarbodiimide (DCC) in DMSO. The DOTA : NHS : DCC molar ratio was 1 : 1.4 : 0.8. The mixture was allowed to react overnight with stirring, and was then filtered to remove the by-product dicyclohexylurea. 1,4,7,10-tetraazacyclododecane-*N*,*N*′,*N*″,*N*′″-tetraacetic acid was conjugated to dLVT at a molar ratio of 50 : 1 by adding an adequate volume of DOTA-activated ester solution to dLVT dissolved in 0.1 M phosphate buffer (pH 8.0). After an overnight reaction, the expected conjugate (Figure 1

) was purified using a reversed-phase column (Resource RPC 1 ml, particle size 15 *μ*m; Amersham Pharmacia Biotech, Uppsala, Sweden) in an FPLC system (LCC-501 Plus controller, Pharmacia Biotech) coupled with a UV detector (LKB-UV-MII, Pharmacia Biotech, *λ*=280 nm) and a radiodetector (Flow Scintillation Analyser, Radiomatic 150 TR, Packard, Meriden, CT, USA). A linear gradient method was applied using a solution of distilled water with 0.1% TFA (solvent A) and methanol (solvent B). The eluents were delivered at a flow of 3 ml min^−1^ starting from 0% of solvent A to 100% of solvent B over 37 min. In this system, dLVT had a retention time of 9 min but, after the conjugation, only one peak appeared after about 12 min. Unreacted DOTA and the reaction by-products were eluted in the first minute together with the solvent front (Figure 2A

). The required compound DOTA-dLVT was recovered by means of an integrated fraction collector (Frac-100, Pharmacia Biotech), and subsequently analysed by means of a MALDI-TOF mass spectrometry (as was the unconjugated dLVT). The mass spectra were recorded on a Voyager-STR spectrometer with delayed extraction (PerSeptive Biosystems, Foster City, CA 94404, USA) using *α*-cyano-4-hydroxycinnamic acid as matrix (Figure 2B).

### Radiolabelling of DOTA-dLVT with ^111^In

After FPLC purification, DOTA-dLVT was concentrated under argon, and then 37 MBq of ^111^InCl_3_, prebuffered with 0.1 M acetic acid–Na acetate buffer (pH 5.5), was added to 10 *μ*g of DOTA-dLVT (corresponding to 0.007 *μ*mol) and heated at 90°C for 30 min. The radiolabelling yield was checked by FPLC as above. A specific activity of 5.3 GBq *μ*mol^−1^ of DOTA-dLVT was obtained with routine radiochemical yields of more than 95%.

### Binding studies on transfected cells

To determine the LVT, dLVT and DOTA-dLVT affinity constants in comparison with OT, heterologous competition experiments were performed on membranes prepared from monkey kidney COS7 cells transiently transfected by means of electroporation with human OTR, V1a, V2 and V1b cDNAs as previously described (Chini *et al*, 1995a). Briefly, the transfected cells were homogenised with a Dounce glass apparatus, washed twice, and resuspended in binding buffer (50 mM Tris HCl, 5 mM MgCl, pH 7.4). Between 5 and 10 *μ*g of membrane proteins were incubated with a fixed concentration of [^3^H]OT (1–2 nM) for 30 min at 30°C in the presence of increasing concentrations of unlabelled peptides. Nonspecific binding was determined in the presence of 1 *μ*M OT. Bound and free radioactivity were separated by filtration over Whatman GF/C filters presoaked in 10 mg ml^−1^ BSA. The binding isotherms were analysed using GraphPad Prism.

### Internalisation assays

HEK293 cells stably transfected with the human OTR fused to EGFP were grown in six-well dishes, washed twice in serum-free medium, and left to equilibrate for 30 min at 37°C. Peptides were then added at a final concentration of 10^−6^ M. At fixed time intervals (5, 15, 30 and 60 min), the cells were washed twice with 10 mM sodium phosphate buffer, pH 7.4, containing 150 mM NaCl (LS buffer), fixed for 20 min at room temperature with 4% (w v^−1^) paraformaldehyde, and mounted on glass slides with 90% (v v^−1^) glycerol in PBS. The slides were observed under an MRC1024 BioRad confocal microscope.

### Binding studies on tumour cells

Human breast carcinoma (MCF7), glioblastoma (MOG-U-V-W) and the KATO human gastric carcinoma cell lines were purchased from ATCC (Manassas, VA, USA). The mammary carcinoma cell line TS/A growing in Balb/c mice was a gift from Professor Guido Forni, University of Turin, Italy. All of the cells were grown as monolayers in RPMI medium (Gibco, Paisley, Scotland) with 10% fetal calf serum (Gibco) in 25 cm^2^ T flasks in a 5% CO_2_ humidified atmosphere at 37°C. Previous experiments have shown that MCF7, MOG-U-V-W and TS/A cells are OTR positive (Cassoni *et al*, 1994, 1998); the KATO cells acted as an OTR-negative control.

The binding experiments were performed on intact cells. Briefly, 20 × 10^5^ cells suspended in 100 *μ*l of culture medium were incubated for 30 min at 4°C in the presence of [^111^In]-DOTA-dLVT 1 *μ*M. The cell suspension was centrifuged for 5 min at 2000 **g**, and the pellet was washed and resuspended in fresh medium. The centrifugation and cell washing were repeated twice. The amount of radiolabelling was evaluated by measuring cell-bound radioactivity (cpm/10^5^ cells) using a Packard auto-gamma counter.

Binding specificity was determined by evaluating radioactive displacement. Briefly, the cell suspension was incubated for 5 min in the presence of LVT, dLVT or OT 100 *μ*M and 1 mM, or in the presence of unrelated peptides (somatostatin 100 nM and 1 *μ*M, and hexarelin 1 *μ*M) followed by 20 min of incubation with 1 *μ*M [^111^In]-DOTA-dLVT. The cells were then centrifuged and washed twice as described above. The activity of the two different agonists and the unrelated peptides in competing with the radioligand was evaluated by measuring cell-bound radioactivity. All of the experiments were performed in triplicate. The statistical analysis was carried out using ANOVA, with a significance cutoff point of 0.05.

### *In vivo* studies in mice

To determine the amount and specificity of the receptor-mediated uptake of [^111^In]-DOTA-dLVT in tumours, we used an experimental model of OTR+ TS/A tumours growing in Balb/c mice. A total of 10 female Balb/c mice weighing 25 g were subcutaneously injected with TS/A mammary carcinoma (0.3 × 10^6^ in 0.2 ml of medium). After 20 days, when the growing tumour had reached about 2 cm in diameter, five mice were intravenously administered 50 *μ*g of cold OT 30 min before the radiotracer and the other five were not. Mice were injected in the tail vein with [^111^In]-DOTA-dLVT (average activity: 74 kBq) and killed 5 h later. The organs were collected, washed, weighed and their radioactivity was measured in a gamma-ray detector with well-counter geometry (Silena, Milan, Italy) together with standards of the injection mixture. Activity was expressed as the percentage of the injected dose (%ID) g^−1^ of tissue, and the uptake ratios of tumour, blood, kidney and liver were calculated.

## RESULTS

### Preparation of DOTA-dLVT and radiolabelling

dLVT (Rimpler, 1971) is characterised by the presence of only one free amino group at position 8. As described above, an activated ester of DOTA obtained via the NHS/DCC system was reacted overnight with dLVT. After purification by means of a reversed-phase column in an FPLC system, we obtained only one peak corresponding to the desired compound: the peak of the unconjugated dLVT (9 min) disappeared after conjugation, and another peak was detected at 12 min (Figure 2A). MALDI-TOF analysis confirmed that the desired dLVT and DOTA-dLVT structures had been successfully obtained (dLVT, [M+H]^+^ 1008.5; DOTA-dLVT, [M+H]^+^ 1393.7; Figure 2B). Radiochemical yields of routinely more than 95% led to [^111^In]-DOTA-dLVT with a specific activity of 5.3 GBq *μ*mol^−1^.

### Affinity of LVT, dLVT and DOTA-dLVT for human OT receptors

LVT, dLVT and DOTA-dLVT affinities for the human OTR were determined by means of heterologous competition experiments on membranes prepared from transiently transfected COS7 cells using [^3^H]-OT as radioligand. As shown in Table 1

**Table 1:** Peptides inhibition constants (*K*_i_ in nM)

	**Human OTR**	**Human V1a**	**Human V1b**	**Human V2**
LVT	2.40±0.425	30.8±4.2	24.9±7.3	2593±625
dLVT	3.88±0.585	35.5±6.2	71.7±2.0	3737±400
DOTA-dLVT	1.21±0.199	370±58	640±177	3562±280

**Figure 3:**
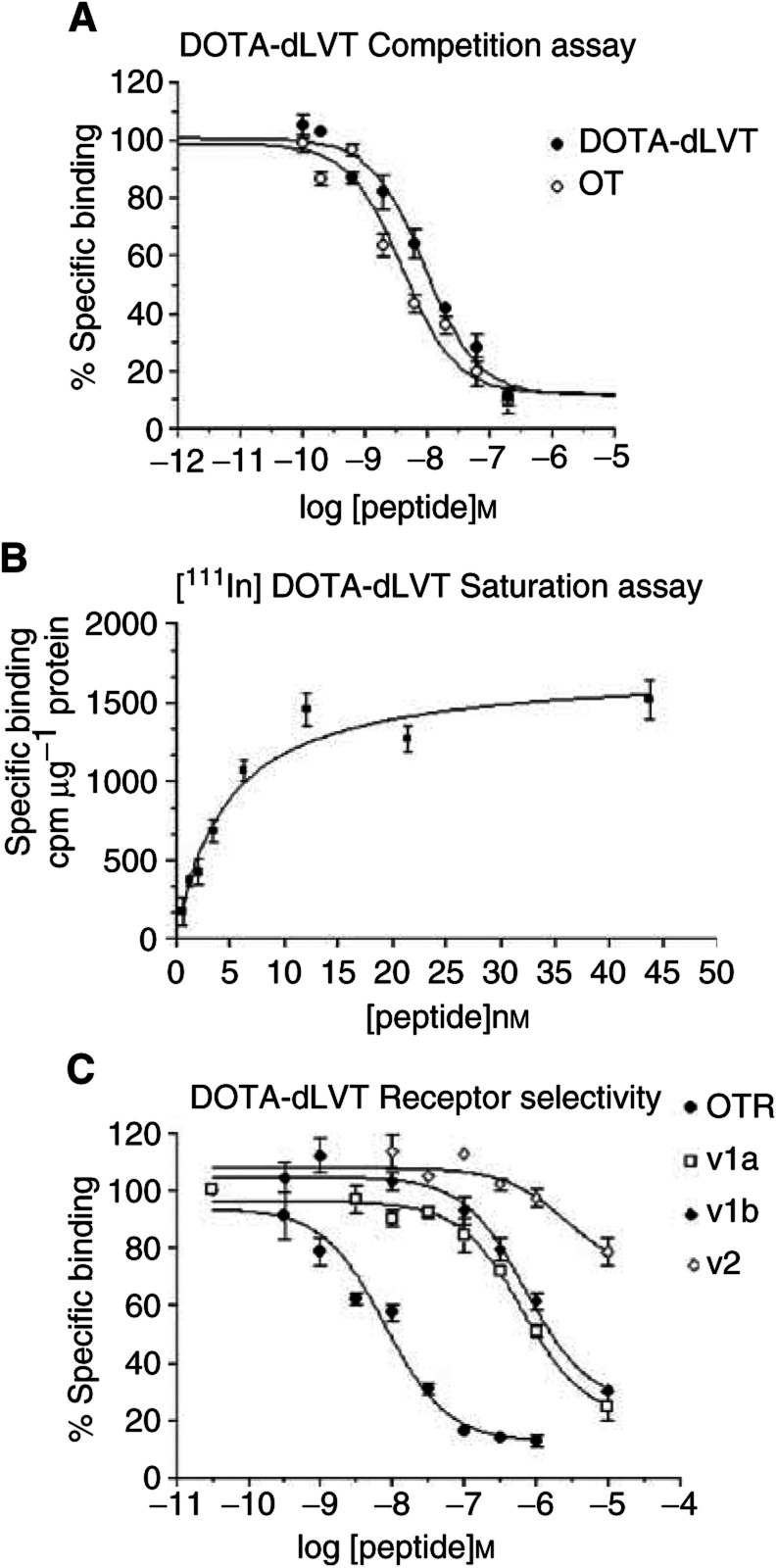
Binding of LVT-derived peptides to human OT/AVP receptor subtypes. (**A**) Comparison of OT and DOTA-dLVT affinity for the human OTR. Competition experiments were performed using membrane preparations of COS7 cells transiently transfected with the wild-type OTR. [^3^H]-OT was used at a concentration of 1–2 nM, and nonspecific binding was determined in the presence of 100 mM OT. Data are expressed as the percentage of bound [^3^H]-OT in the absence of cold competitor. The curves are representative of three independent assays performed in triplicate. (**B**) [^111^In]-DOTA-dLVT saturation binding assay. The affinity of [^111^In]-DOTA-dLVT for the human OTR was measured by incubating increasing amounts of labelled peptide to membrane preparations of COS7 cells transiently transfected with the wild-type OTR. The curve is representative of two independent assays performed in triplicate. (**C**) Receptor selectivity profile of DOTA-dLVT. Competition experiments were performed using membrane preparations of COS7 cells transiently transfected with the wild-type human OTR, and V1a, V1b and V2 receptors. The membrane preparations of OTR-expressing cells were incubated in the presence of a constant amount of ^3^[H]OT (1–2 nM), and nonspecific binding was determined in the presence of 100 *μ*M OT. The membrane preparations of cells expressing V1a, V1b and V2 receptors were incubated in the presence of a constant amount of ^3^[H]AVP (2–4 nM), and nonspecific binding was determined in the presence of 100 *μ*M AVP. The data are expressed as percentages of bound [^3^H]-ligand in the absence of cold competitor. Each curve is the mean of triplicate determinations of a single representative experiment.

Binding of LVT-derived peptides to human OT/AVP receptor subtypes. Competition experiments were performed using membrane preparations of COS7 cells transiently transfected with the wild-type human OTR, and V1a, V1b and V2 receptors. The *K*_i_ values for the human OTR were obtained by displacement of [^3^H]-OT; the *K*_i_ values for the human V1a, V1b and V2 receptors were obtained by displacement of [^3^H]-AVP. The values are the mean values±s.e. of three different experiments each performed in triplicate.

, unlabelled LVT, dLVT and DOTA-dLVT inhibited [^3^H]-OT binding with very similar *K*_i_ values. As shown in Figure 3A

, the OT and DOTA-dLVT displacement curves were parallel and their slopes were not significantly different.

[^111^In]-DOTA-dLVT affinity for human OTR was also measured on membranes prepared from transiently transfected COS7 cells by means of saturation binding assays. As shown in Figure 3B, the binding of [^111^In]-DOTA-dLVT was saturable with a calculated *K*_d_ of 4.5 nM, which was very similar to the *K*_i_ value of unlabelled DOTA-dLVT calculated from the competition experiments.

Our binding experiments therefore indicated that the addition of a single DOTA-group at position 8 of dLVT brought about a 2–3-fold enhancement of affinity for the hOTR. Furthermore, the binding properties of DOTA-dLVT remained unchanged after labelling with [^111^In].

### Receptor selectivity

OT/AVP analogues bind to and activate (albeit with different affinities and efficacies) all members of the OT/AVP receptor family, which includes the OTR and the three vasopressin receptor subtypes V1a, V1b and V2 (Peter *et al*, 1995; Chini *et al*, 1995b; Barberis *et al*, 1999). When developing a specific OTR analogue, it is therefore essential to check its binding properties not only on the human OTR, but also on the V1a, V1b and V2 subtypes. To this end, receptor specificity was analysed by comparing the affinities of LVT, dLVT and DOTA-dLVT in cells transfected with all of the members of the OT/AVP receptor family. As shown in Table 1, all three compounds preferentially bound the human OTR, the only receptor subtype capable of binding them with nanomolar affinity. The V1a and V1b receptors showed a 10-fold lower affinity for LVT and dLVT, and the V2 receptor bound them only at micromolar concentrations. Interestingly, OTR selectivity further increased with the addition of the DOTA group. As shown in Table 1 and Figure 3C, all of the vasopressin subtypes challenged with DOTA-dLVT showed at least a 300-fold decrease in binding affinity. These data indicate that DOTA-dLVT not only binds the human OTR with very high affinity, but also exhibits a strikingly high selectivity for the human OTR relative to the vasopressin V1a, V1b and V2 receptor subtypes.

### Peptide-induced receptor internalisation

**Figure 4:**
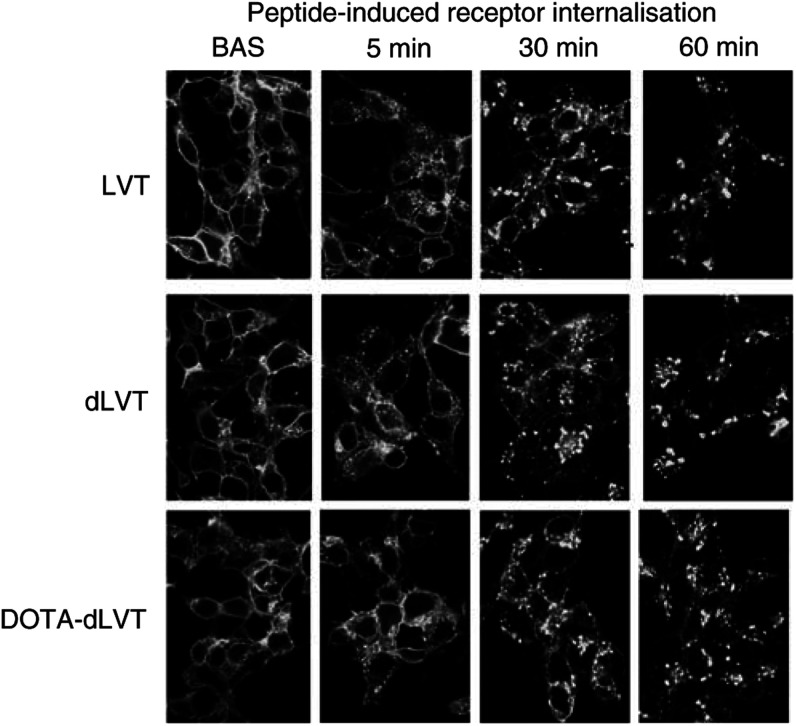
Laser-scanning confocal microscopy analysis of peptide-induced receptor internalisation. Receptor internalisation was investigated in HEK293 cells stably transfected with the human OTR bearing the EGFP fused at its C-terminus. The cells were grown on coverslips and incubated in serum-free medium for 30 min (Bas), after which the peptides were added at a final concentration of 10^−6^ M and the coverslips were fixed at 1, 5, 15, 30 and 60 min.

To assess whether LVT, dLVT and DOTA-dLVT behave like OT to induce receptor internalisation, we applied them to HEK293 cells stably expressing a human OTR C-terminally tagged with the green fluorescent protein EGFP (Guzzi *et al*, 2002; Rimoldi *et al*, 2003). As shown in Figure 4

, under basal conditions, the fluorescent receptor is mainly present at the cell surface. However, punctate fluorescence (presumably associated with endocytotic vesicles) could be seen as early as 5 min after the application of LVT, dLVT and DOTA-dLVT. After 30 and 60 min, the fluorescent patches appeared to be mainly localised in intracellular compartments. These data indicate that DOTA-dLVT is capable of inducing a persistent internalisation of the human OTR.

### [^111^In]-DOTA-dLVT binding to OTR+ and OTR− tumour cells

The ability of DOTA-dLVT to bind to OTR expressed in cancer cells was investigated by means of a 30 min incubation with [^111^In]-DOTA-dLVT, after which the presence of specific radiolabelling was observed in all three OTR-positive cell lines (MCF7, TS/A and MOG-U-V-W), but was negligible in the OTR-negative HT29 cell line. Expressed as cpm/10^5^cells, the amount of labelling was 1201±76 for MCF7, 7120±24 for TS/A and 1523±61 for MOG-U-V-W, and only 80±6 for KATO. The specificity of the binding was proved by means of displacement with cold radioligands: more than 92% of the specific [^111^In]-DOTA-dLVT binding in OTR-positive cells was displaced by 5 min of preincubation with 100 *μ*M and 1 mM nonradioactive OT or LVT.

### Preclinical study in animals bearing OTR+ tumours

Biodistribution studies showed that [^111^In]-DOTA-dLVT was capable of binding *in vivo* to OTR in TS/A tumours (Table 2

**Table 2:** Preclinical study of animals bearing TS/A tumours

	**Group I [^111^In]-DOTA-dLVT and cold OT**	**Group II [^111^In]-DOTA-dLVT only**
Tumour (%ID g^−1^)	0.079	0.230
Blood (%ID g^−1^)	0.017	0.030
Kidney (%ID g^−1^)	1.693	3.559
Liver (%ID g^−1^)	0.075	0.162
Tumour/blood ratio	4.54	7.58
Tumour/kidney ratio	0.05	0.06
Tumour/liver ratio	1.05	1.42

Balb/c mice bearing TS tumours positive for the human OTR were intravenously injected with [^111^In]-DOTA-dLVT. Five mice were injected with 50 *μ*g of cold OT 30 min before receiving the radiotracer (Group I); five mice received only the radiotracer (Group II). A 20-fold dilution of the injected dose (average activity 74 kBq) was used as a standard for the total administered activity. Activity is expressed as a percentage of injected dose (%ID) g^−1^ of tissue, and ratios of uptake between tumour, blood, kidney and liver were calculated. The data are averages of five replicates; the activity remaining in the tail was also considered and subtracted from the total activity for the calculations.

). The specific uptake in tumour (0.23%ID g^−1^) was higher than that in blood (0.03%ID g^−1^) or liver (0.16%ID g^−1^), thus leading to tumour/blood and tumour/liver uptake ratios of, respectively, 7.58 and 1.42. As expected, kidney showed the highest uptake (3.56%ID g^−1^). When the TS/A tumours were blocked with 50 *μ*g of cold OT, there was a three-fold decrease in the uptake of [^111^In]-DOTA-dLVT.

## DISCUSSION

We report here the synthesis and pharmacological properties of a new receptor-mediated radiotracing compound ([^111^In]DOTA-dLVT), specific for OTR-expressing cells and tumours.

As a starting compound, we used dLVT (Rimpler, 1971), an OT analogue with only one potential binding site for a chelating agent: the free *ɛ*-NH_2_ group of the lysine residue in position 8. The lysine *ɛ*-NH_2_ group has been widely used to link chemical reagents to peptides (Meares *et al*, 1984; Terrillon *et al*, 2002). We used a slight molar excess of DOTA to NHS/DCC in order to activate only one carboxyl group and prevent DOTA from bridging two or more dLVT molecules. After linking DOTA to dLVT, no polymeric compounds were detected by MALDI-TOF mass spectrometry analysis, thus confirming the 1 : 1 binding of the chelating agent to the only active binding site.

Purified DOTA-dLVT showed a very high (nanomolar) affinity for the human OTR, analogous to that exhibited when the fluoresceinyl (Flu) group was attached to the Lys8 residue in dLVT to give d[Lys^8^(5/6 C-Flu)]VT (Terrillon *et al*, 2002). Deamination of OT and its analogues had been shown to enhance biological activity *in vivo* by making the peptide less susceptible to aminopeptidase (Golubow and du Vigneaud, 1963).

Since OT/vasopressin analogues bind to and activate four different receptor subtypes (OTR, V1a, V1b and V2) (Chini *et al*, 1995b; Peter *et al*, 1995; Barberis *et al*, 1999), peptides of potential clinical use should be checked for their receptor selectivity. Our competition experiments on receptor-rich membrane preparations showed that dLVT binds to human OTR, V1a, V1b and V2 with a pharmacological profile that is very similar to that of OT. Interestingly, the addition of the DOTA moiety in position 8 of dLVT brought about a 2–3-fold increase in affinity for the human OTR, compared to both OT and dLVT, while greatly reducing its affinity for the V1a, V1b and V2 receptor subtypes. These findings closely agree with those of a previous study based on molecular modelling and site-directed mutagenesis, which indicated that the interactions of the residue in position 8 are crucial in determining the high affinity of OT/AVP peptides for the vasopressin receptor subtypes (Chini *et al*, 1995a), but are much less important for the human OTR (Chini *et al*, 1995b). The striking increase in receptor selectivity of DOTA-dLVT for the human OTR is particularly relevant because it should reduce the likelihood of side effects due to peptide binding to the V1a, V1b and V2 vasopressin receptors.

As a chelator, DOTA is sufficiently flexible to be efficiently radiolabelled with not only diagnostic *γ*-ray emitters such as ^111^In, but also with therapeutic *β*^−^ radionuclides (e.g. ^90^Y, *E*_*β*max_=2.28 MeV, range 1.1 × 10^−2^ m, and ^177^Lu, *E*_*β*max_=0.50 MeV, range 2 × 10^−3^ m) as it has already been successfully used to convey them to human tumours in association with somatostatin analogues (Otte *et al*, 1998; Paganelli *et al*, 1999). We found that DOTA-dLVT was very efficiently labelled with ^111^In and we are currently investigating its labelling with therapeutic radionuclides.

To be used in therapeutic applications, a radiopeptide should not only have a binding profile of high affinity and selectivity, but must also be characterized by persistent presence at tumour sites. In the case of analogues that bind to membrane receptors, one very useful property is their ability to enter cells as a result of peptide-induced receptor internalisation, which would have a number of positive effects: the radionuclide would remain longer at the tumour site and there would not be a decrease in surface binding due to peptide dilution. Furthermore, the internalised peptide would be closer to the DNA (the final target of the cytotoxicity exerted by radionuclides such as [^90^Y]) and, as [^90^Y] emits short-range Auger electrons, the surrounding cells would remain unaffected. Using a fluorescent reporter (EGFP) constitutively linked to OTR and stably transfected in HEK293 cells (Rimoldi *et al*, 2003), we traced the fate of the receptor after LVT, dLVT and DOTA-dLVT binding. All of these compounds induced a rapid and persistent receptor internalisation similar to that induced by oxytocin (Guzzi *et al*, 2002). Given that the binding of the radioactive metal to the chelating agent does not affect its affinity and is unlikely to affect the internalisation of the ligand–receptor complex, the tracer will probably remain inside the target and thus ensure an enhanced tumour/blood ratio allowing efficient imaging and cytotoxicity. Our *in vitro* and *in vivo* murine experiments showed that ^111^In-DOTA-dLVT selectively binds to OTR-positive cells and tumours, and is thus a potentially powerful reagent for the imaging and treatment of OTR-positive cancers. As OTRs are expressed in various tumours in which conventional chemo- and hormonal therapy may slow disease progression but are not curative (particularly brain glioblastomas and metastatic breast cancers), radiolabelled DOTA-dLVT offers an exciting and entirely new approach to the treatment of such life-threatening malignancies.
